# Establishment and characterization of 38 novel patient-derived primary cancer cell lines using multi-region sampling revealing intra-tumor heterogeneity of gallbladder carcinoma

**DOI:** 10.1007/s13577-021-00492-5

**Published:** 2021-04-04

**Authors:** Feiling Feng, Qingbao Cheng, Bin Li, Chen Liu, Huizhen Wang, Bin Li, Xiaoya Xu, Yong Yu, Zishuo Chen, Xiaobing Wu, Hua Dong, Kaijian Chu, Zhenghua Xie, Qingxiang Gao, Lei Xiong, Fugen Li, Bin Yi, Dadong Zhang, Xiaoqing Jiang

**Affiliations:** 1 Department of Biliary I, Shanghai Eastern Hepatobiliary Surgery Hospital, Navy Military Medical University, Shanghai, 200438 China; 2 3D Medicines Inc., Shanghai, 201114 China

**Keywords:** Gallbladder carcinoma, Genomic profiling, Intra-tumor heterogeneity, Patient-derived primary cancer cell line, Transcriptome profiling

## Abstract

Gallbladder carcinoma (GBC) is a lethal biliary tract malignant neoplasm. Patient-derived primary cancer cell lines (PDPCs) are appropriate models to explore biological characteristics and potential therapeutics; however, there is a lack of PDPCs in GBC. In this study, we aimed to establish and characterize the GBC PDPCs, and further investigated the intra-tumor heterogeneity (ITH). Multi-region sampling (3–9 regions) of the operable tumor tissue samples was used to establish PDPCs. Short tandem repeat genotyping for cell authentication and karyotyping was performed, followed by whole-exome sequencing and RNA sequencing to assess the ITH at the genetic and transcriptional levels, respectively. Thirty-eight PDPCs were successfully established from seven GBC patients and characterized. ITH was observed with a median of 38.3% mutations being heterogeneous (range, 26.6–59.4%) across all patients. Similar with other tumor types, *TP53* mutations were always truncal. In addition, there were three genes, *KMT2C*, *CDKN2A*, and *ARID1A*, with truncal mutations in at least two patients. A median of 370 differentially expressed genes (DEGs) was identified per patient. Distinct expression patterns were observed between major histocompatibility complex (MHC) class I and II genes. We found the expression of MHC class II genes in the PDPC samples was closely regulated by *CIITA*, while that of MHC class I genes were not correlated with *CIITA* expression. The PDPCs established from GBC patients can serve as novel in vitro models to identify the ITH, which may pave a crucial molecular foundation for enhanced understanding of tumorigenesis and progression.

**Supplementary Information:**

The online version contains supplementary material available at 10.1007/s13577-021-00492-5.

## Introduction

Gallbladder carcinoma (GBC) is one of the lethal biliary tract cancers with limited therapeutic options and unsatisfactory treatments [1, 2]. Surgical resection is the preferred treatment option, but many GBC patients are not suitable for curative surgery when they are detected [3, 4]. Patients with advanced or metastatic GBCs have a poor prognosis with a 5-year survival rate of less than 10% [5, 6]. Among all the potential reasons, the lack of understanding regarding the tumorigenesis and progression has been proposed as a major obstacle to discovering a new strategy for more precise and effective treatment to improve GBC patients’ prognosis.

Several previous studies of GBCs have reported the results of mutational profiling [7–13]. The recurrently mutated actionable genes and the signaling pathways in GBCs were described, which may be regarded as the potential candidates for personalized targeted therapy. Actually, treatment development and improvement may require not only appreciation of genetic alterations but also understanding of intra-tumor heterogeneity (ITH) [14], which is a well-established phenomenon and may foster tumor adaptation, and drug resistance to chemotherapy and molecularly targeted agents [15]. Some studies have demonstrated the universal prevalence of ITH in many types of tumors including renal-cell carcinoma, lung adenocarcinomas and glioblastoma [16–18]. However, little is known about the ITH and its impact on the progression of GBC.

Patient-derived primary cancer cells (PDPCs) derived from tumor tissue samples are not only attractive for testing drug sensitivity and exploring the biological functions, but also preferable for the tumor features using genomic sequencing [19]. Moreover, previous studies on hepatocellular carcinoma and intrahepatic cholangiocarcinoma (ICC) have shown that PDPCs can enable an accurate assessment of ITH [14, 19]. In the present study, we established thirty-eight PDPCs using multi-region sampling and took advantage of them combined with whole-exome sequencing (WES) and RNA sequencing (RNA-seq) to explore the ITH in GBC.

## Materials and methods

### Patients and clinical samples

Patients with GBCs were enrolled in this study from August 2015 to March 2016. All patients provided fresh tumor tissue samples that were obtained from the clinical sample bank at the Department of Biliary I, Shanghai Eastern Hepatobiliary Surgery Hospital, Navy Military Medical University, and samples were collected. All the patient was diagnosed by surgical pathology. Clinical information of these patients is shown in Table 1. Based on the seventh edition of the American Joint Committee on Cancer (AJCC) staging system for esophageal cancer [20], one patient was stage IIa, four patients were stage IIIa, and two patients were stage IIIb. All patients provided written informed consent for their samples to be examined and their clinical data to be utilized. This study was approved by the Institutional Review Board of Shanghai Eastern Hepatobiliary Surgery Hospital, Navy Military Medical University (No. EHBHKY2015-02-010).

**Table 1:** Clinical characteristics of seven patients with gallbladder carcinoma

Patient_ID	Pathological diagnosis	Sex	Age	Site	Size (cm)	AJCC Tumor Stage
668	Gallbladder carcinoma	Male	60	Gallbladder	3	IIIb
902	Gallbladder carcinoma	Female	60	Gallbladder	10	IIIa
1279	Gallbladder carcinoma	Male	49	Gallbladder	11	IIIa
1405	Gallbladder carcinoma	Male	65	Gallbladder	4	IIIa
1436	Gallbladder carcinoma	Male	65	Gallbladder	4.2	IIa
4160	Gallbladder carcinoma	Female	46	Gallbladder	3.5	IIIb
4256	Gallbladder carcinoma	Female	78	Gallbladder	6.3	IIIa

### Establishment and culture of PDPCs

The spatially distinct multi-regions of the operable GBC tissue samples were collected from seven patients and PDPCs were established by following the standard procedures. In brief, fresh tissues were washed with sterile phosphate-buffered saline (PBS) and cut into 1 mm^3^ pieces. Then, small pieces of the tissue were placed into 10 cm^2^ dishes and incubated with DMEM/F12 medium with 10% fetal bovine serum (FBS) (Gibco, Thermo Fisher Scientific, Inc., Waltham, MA, USA), 1% non-essential amino acids, and 1% penicillin/streptomycin (Thermo Fisher Scientific, Inc., Waltham, MA, USA). The cells were trypsinized with 0.25% trypsin (Gibco, Thermo Fisher Scientific, Inc., Waltham, MA, USA) when they reached about 30% confluence and were transferred into 24-well plates for subculture. Thereafter, all PDPCs were grown in a 37 °C incubator at 5% CO_2_ with DMEM/F12 medium supplemented with 10% FBS. The mycoplasma testing has been done for all PDPCs established in this study. The passage of each PDPC used in the following experiment was different and the passage information for each PDPC in the assays ranged between 15 and 25.

### Characterization of PDPCs

For short tandem repeat (STR) genotyping, genomic DNA was extracted from each GBC PDPC using a QIAamp DNA Mini Kit (QIAGEN Inc., Valencia, CA, USA). Nineteen STR loci (TH01, D12S391, D7S820, CSF1PO, FGA, D5S818, D2S1338, D21S11, D18S51, TPOX, vWA, D8S1179, D3S1358, D13S317, D6S1043, D16S539, Penta E, D19S433, and Penta D) and Amelogenin were amplified by PCR and analyzed using an Applied Biosystems 3730xl DNA Analyzer (Applied Biosystems Inc., Foster City, CA, USA). For karyotyping, exponentially growing GBC PDPCs were exposed to colchicine (0.01 mg/ml) for 16 h and then to hypotonic treatment (0.075 mol/L KCl) for 20 min. After fixation in a methanol and acetic acid mixture (3:1 by volume), cell suspensions were dropped onto ice-cold slides. Slides were then treated in trypsin for 30–60 s and stained with Giemsa. Chromosomes from at least 20 metaphases per sample were analyzed under a microscope.

### WES and RNA-seq

All genomic DNA and total RNA samples were shipped on dry ice after extraction, and WES was performed as previously described [21]. DNA library preparation, and sequence capture and next-generation sequencing were performed at WuXi Next CODE (Shanghai, China). Deep sequencing of exome captured DNA was performed on an Illumina NovaSeq S4 PE150 instrument.

RNA-seq was performed as previously described [22]. Randomly interrupted mRNA and the first cDNA strand were synthesized, and then a second cDNA strand was synthesized. RNA-seq library construction and hybrid capture and sequencing were performed at WuXi Next CODE (Shanghai, China). RNA-seq was performed using an Illumina HiSeq X Ten PE150 instrument.

### Somatic single-nucleotide variations (SNVs) and insertions and deletions (INDELs) calling

For DNA data, raw sequencing reads were aligned to the human reference genome hg19 using BWA-MEM [23] (version: bwa-0.7.12) with default settings. Mapped reads in the SAM format were then sorted and converted to BAM format using Picard (version 2.0.1, https://broadinstitute.github.io/picard/), followed by the duplicate removal process. Sequencing QC metrics, such as mean target coverage, duplication rate, on target rate and insert size, were calculated using an in-house script. SNVs and INDELs were detected using MuTect [24] (version: mutect-1.1.7) and Pindel [25] (version 0.2.5a8), respectively. The SNVs and INDELs were then combined together as the initial variant calls for each sample. The initial variant calls were then annotated using ANNOVAR [26] (Version Date: 2015-04-24). Somatic variants were selected based on the following criteria: (1) sites with strand bias ≥ 0.9 were removed; (2) for a given site, the total coverage ≥ 20 and reads supported the alternative allele ≥ 8; (3) variant allele frequency ≥ 0.1; (4) common single-nucleotide polymorphisms (SNPs) and INDELs in dbSNP (AF ≥ 1%) were removed; (5) common SNPs from a Chinese population (CONVERGE, https://www.biorxiv.org/content/biorxiv/early/2017/07/13/162982.full.pdf, https://doi.org/10.1101/162982) were removed; (6) INDELs from the 1000 Genome Project [21] gold standard indels were removed; (7) sites with AF larger than 0.015 in any of the three databases (1000 Genome Project [27], ESP6500 [28], and ExAC [29]) were removed; and 8) exonic or splicing sites were selected. Known mutations in the latest COSMIC [30] database were marked using BEDTools [31] (version: 2.24.0).

### Phylogenetic analysis and ITH estimation

In each individual, both non-silent and silent somatic mutations occurred in at least one sample were used to construct the phylogenetic tree. A binary presence/absence matrix was generated according to the occurrence of mutations across all samples in each individual. Phylogenetic trees were built across all individuals based on the binary matrix using the Penny program in the PHYLIP package (version 3.679, http://evolution.genetics.washington.edu/phylip.html) with the parsimony ratchet method. Trees were manually reconstructed; thus, making the trunk/branch lengths scaled in proportion to the number of mutations. For each individual, variants were classified as trunk, shared and private mutations if they were identified ubiquitously across all the PDPC samples, in more than one but not all samples, and in only a single sample, respectively. For the convenience of description, shared and private mutations together were further classified as non-trunk mutations. To estimate the ITH for each individual, a percentage was calculated as the number of trunk mutations divided by the number of non-trunk mutations.

### Identification of putative driver mutations and analysis of mutational signatures

To identify driver mutations in GBC patients, a list of 573 candidate genes was collected from the COSMIC cancer gene census (December 2018) and recent large-scale GBC and cholangiocarcinoma (CCA) sequencing studies [7, 32–34]. Putative driver mutations were then identified from variants in these genes if they met one of the following criteria: (1) predicted to be nonsense, splicing, or frameshift mutations and (2) predicted to be deleterious in at least one of the SIFT and PolyPhen programs (annotated using ANNOVAR) for missense mutations.

To extract the composition of mutational signatures in each PDPC, deconstructSigs [35] was performed on somatic SNVs (both synonymous and non-synonymous) for trunk and non-trunk mutations separately. Mutational signatures were based on the Wellcome Trust Sanger Institute Mutational Signature Framework [36]. Overall distribution of mutational signatures was compared between trunk and non-trunk mutations within each individual.

### Identification of somatic copy number variations (CNVs)

To identify CNVs in each PDPC, CNVkit [37] (version: v0.8.5) with default parameters was run on the mapped reads. A Panel of Normal (PON) was built as a normal control using a set of 10 blood samples from other individuals with the same library method. The initial copy number profile of each PDPC was derived using log2 ratio of read coverage between the tumor and the PON by each segment. Segments located on chromosomes X and Y were excluded from further analysis. Segment results in log2 ratio of all samples were combined together to identify regions that were significantly amplified or deleted across our cohort using GISTIC2.0 [38]. The GISTIC2.0 command line was as follows: *gistic2 -b output –seg samples.seg –mk samples.marker -refgene hg19.mat -genegistic 1 -smallmem 1 -broad 1 -brlen 0.8 -conf 0.99 -ta 0.3 -td 0.3 -qvt 0.01 -armpeel 1 -savegene 1 -gcm extreme*. The GISTIC2.0 peak regions at the cytoband level were then selected to examine ITH in CNVs.

### Gene expression profiling

Raw reads from RNA-seq were mapped to the human reference genome hg19 using STAR [39] (version: v2.5.2b) with default parameters. RSeQC [40] (version 2.4) was run on the mapped reads to visualize the read distribution over genome features, such as CDS exon, 5′UTR exon, 3′ UTR exon, Intron, and Intergenic regions. Raw counts of each gene in RefSeq (February 2017) for each sample were calculated using featureCounts [41] (version 1.5.2). The expression profile of each sample at the count level was normalized to reads per kilo base per million mapped reads (RPKM) using an in-house R script. All profiles were combined together as an integrated expression matrix for further analysis. To investigate the ITH at the gene expression level for each GBC patient, lowly expressed genes (average log_2_RPKM < 1) were first removed within each patient. Next, the most varied genes (median absolute deviation log_2_RPKM >  = 1) across all samples in each patient were selected for further analysis. A hierarchical cluster analysis was performed on these genes and samples were classified into two groups based on the hierarchical tree. Differentially expressed genes (DEGs) were then identified as fold change >  = 2 or <  = 0.5 between the two groups. To visualize ITH in the expression profiles for each patient, a heat map was drawn on these DEGs. To explore the potential biological relevance of these DEGs, Gene Ontology (GO) enrichment analyses were performed using R package ClusterProfiler [42].

### Statistical analysis

The differences between trunk and non-trunk mutations were evaluated using proportions test with continuity correction. *P* < 0.05 was considered statistically significant. Statistical analysis was performed using R software, version 3.5.0 (R Foundation for Statistical Computing).

## Results

### Establishment and characterization of PDPCs

**Fig. 1:**
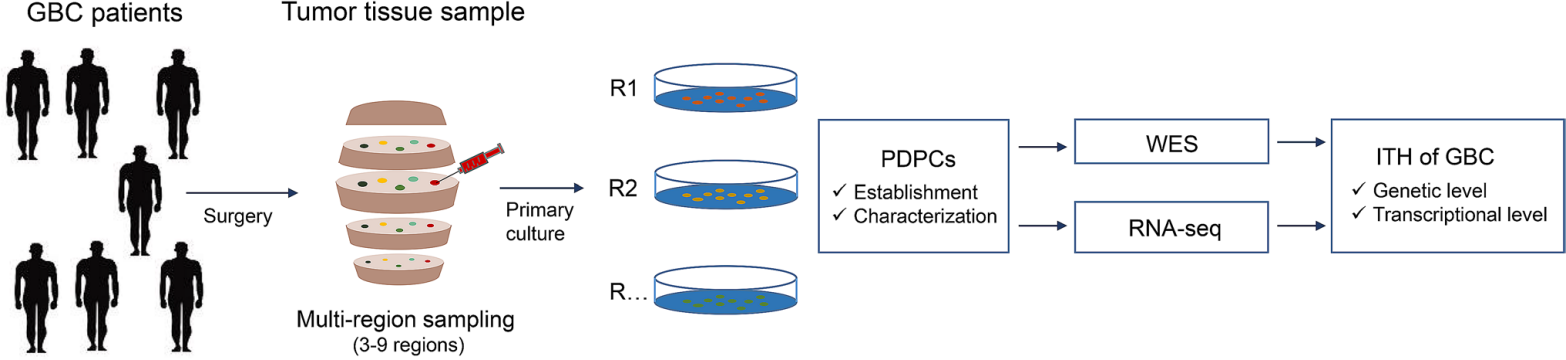
Flowchart of study design. Multiple spatially separated tumor tissue regions (ranging of 3–9 regions) from seven patients with GBC were sampled for primary culture. After the characterization, the established PDPCs were then subjected to WES and RNA-seq analysis for the ITH of GBC at the genetic and transcriptional levels

In this study, we successfully established thirty-eight PDPCs derived from spatially distinct multi-regions of the operable tumor tissue samples of seven GBC patients (Table 1), ranging from three to nine PDPC samples each patient (Fig. 1). To characterize the 38 PDPCs, morphology, karyotype examination and short tandem repeats (STRs) were performed (Supplementary Fig. 1, Supplementary Table 1 and Supplementary Table 2). Furthermore, comparison analyses of STRs of these GBC PDPCs with those from American Type Culture Collection (ATCC) and Deutsche Sammlung von Mikroorganismen und Zellkulturen (DSMZ) suggested that GBC PDPCs did not match those in existing databases, devoid of cross contamination with other known cancer cell lines. In addition, the results of single-nucleotide polymorphisms (SNPs) showed that PDPCs from the same patient were clustered together (Supplementary Fig. 2), which confirmed the origin of these PDPC samples.

### Genomic architecture and ITH of mutations in GBC

**Fig. 2:**
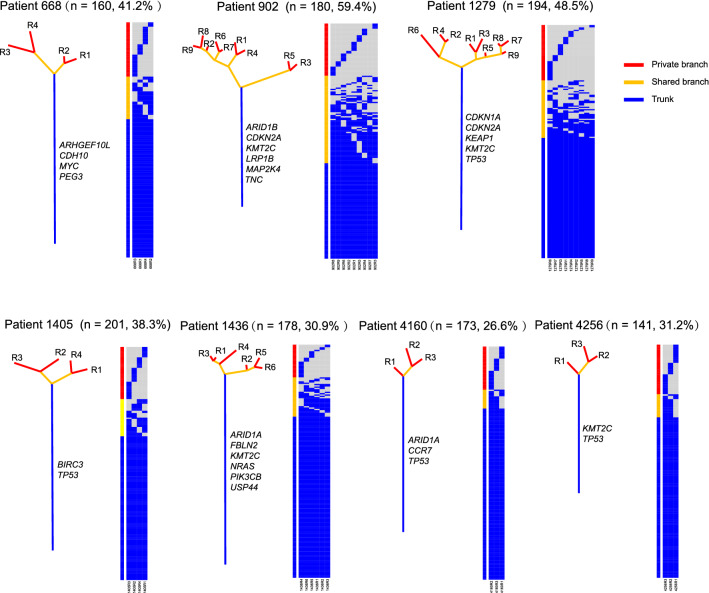
ITH of mutations in seven patients with GBC. Phylogenetic trees were generated from somatic mutations using the parsimony ratchet method, and the branch lengths were scaled in proportion to the number of variants. Heat map nearby each tree showed the occurrence (presence in blue and absence in grey) of each mutation in each patient-derived primary cancer cell line. Genes with putative driver mutations were displayed beside the trunk branch of each individual. The number of mutations and the ITH score were listed on the top of each individual panel. Branches were colored according to the mutation classification: blue: trunk mutations, yellow: shared mutations, red: private mutations

WES was performed on the genomic DNA from 38 PDPCs. A total of thirty-eight PDPCs (range, 3–9 per patient) were sequenced, achieving a median target depth of 136X. A total of 809 non-silent mutations (affecting 734 genes) and 291 silent mutations were identified in these 38 PDPCs. To assess the ITH and clonal evolution in GBC patients, a phylogenetic tree was constructed based on the occurrence of mutations in multiple PDPCs derived from each patients (Fig. 2). The phylogenetic trees exhibited different evolution models across different patients, with a median of 38.3% of variants having spatial heterogeneity (range, 26.6–59.4%). The phylogenetic tree structure varied across GBC patients. For example, the phylogenetic tree of patient 902 had a longer branch than its trunk, whereas the one of patient 4160 displayed a much more homogenous mutational pattern. Notably, patient 902 had the highest ITH in our cohort and two PDPCs (R3 and R5) showed huge big difference compared to the other seven PDPCs. The patients harbored mutations in different candidate driver genes (displayed near the trunk of each phylogenetic tree) in the trunk branch, exhibiting distinct driven patterns across these patients.

### Mutation status of putative driver genes

**Fig. 3:**
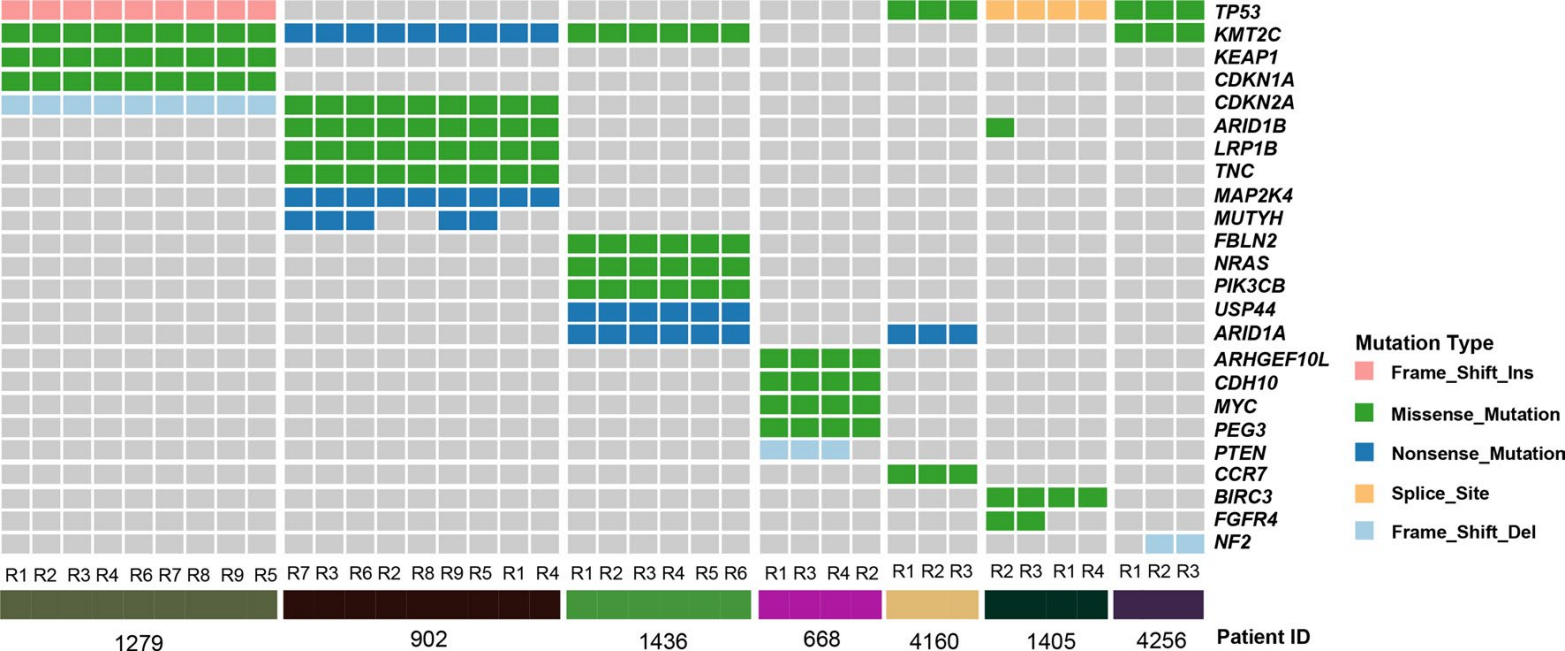
Mutation status of putative driver genes. A heat map displayed the putative driver mutations in each PDPC sample of the patients with gallbladder carcinoma. Mutations were colored according to the variant classifications

To better understand the cancer genome evolution of GBC, a list of 573 manually curated genes was further inspected to identify the potential driver mutations. There were 24 (24/573) genes with putative driver mutations in at least one sample of our cohort, and the majority mutations in these genes were truncal (Fig. 3). Notably, most of these genes (19 out of 24 genes, except *TP53*, *KMT2C*, *CDKN2A*, *ARID1B*, and *ARID1A*) were exclusively mutated in patients, indicating diverse driver events during tumorigenesis in each patient. Consistent with previous studies in other cancer types, *TP53* was the most recurrently mutated gene (4 out of 7 patients) in our cohort and mutations of this gene were always truncal despite distinct mutation types across different individuals. Similarly, *KMT2C* (*MLL3*) had the same mutation frequency as *TP53* and mutations in this gene were also truncal across different patients. There were two genes, *CDKN2A* and *ARID1A*, with truncal driver mutations in two out of seven patients.

Although most mutations were homogeneous across different PDPCs, a few mutations showed heterogeneity. The *ARID1B* mutation was truncal in patient 902 while only one PDPC from patient 1405 harbored the *ARID1B* mutation. A nonsense mutation of *MUTYH* was found in 5 out of 9 PDPCs from patient 902, while a missense mutation of *FGFR4* was identified in 2 out of 4 PDPCs from patient 1405. Meanwhile, a frameshift deletion of *PTEN* and *NF2* was discovered in 3 out of 4 PDPCs from patient 668 and in 2 out of 3 PDPCs from patient 4256, respectively.

### Dissecting mutational spectra and signatures

**Fig. 4:**
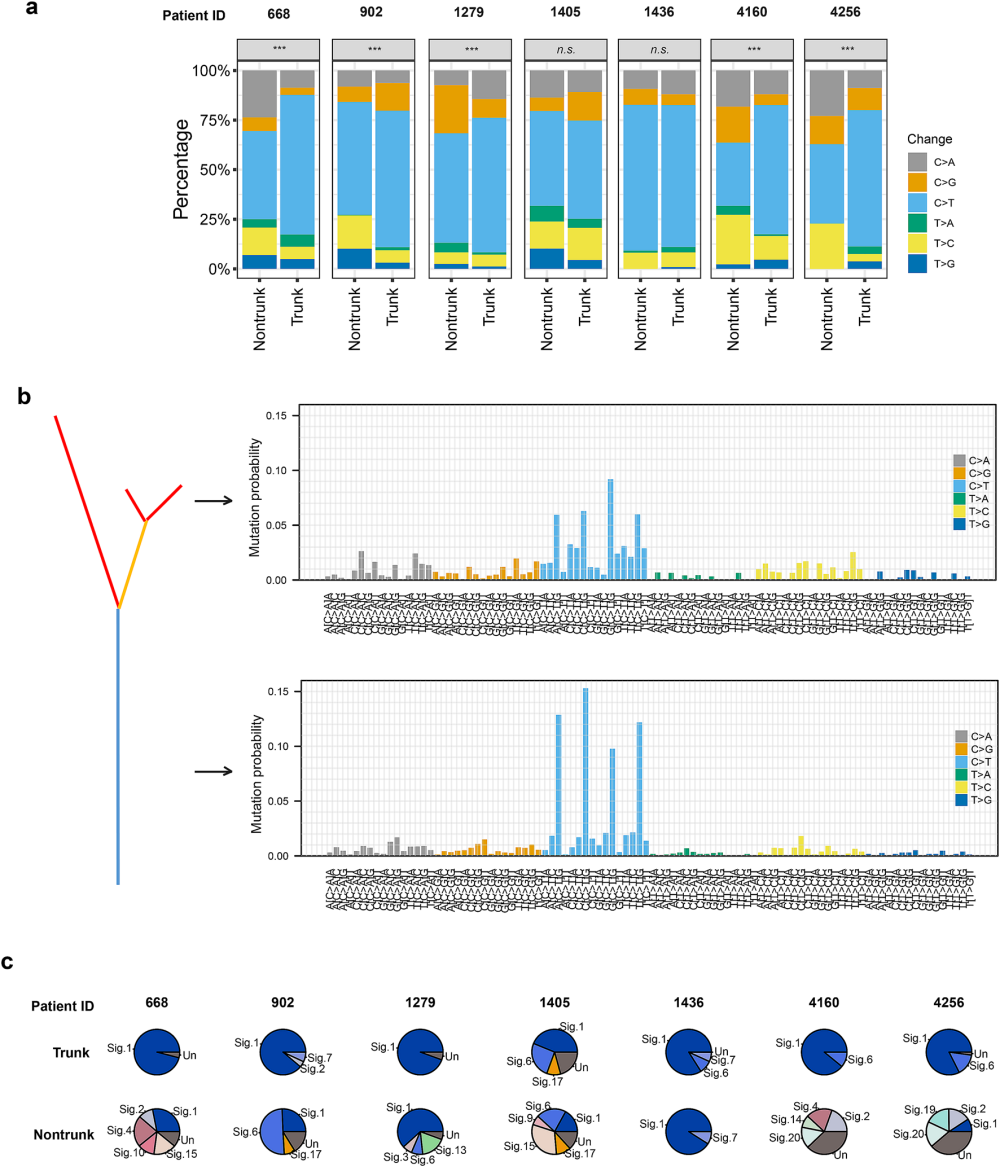
Dissecting mutational spectra and signatures. **a** The distribution of variant changes between trunk and non-trunk mutations. The differences between trunk and non-trunk mutations were evaluated using proportions test with continuity correction. * < 0.05, 0.001 < ** < 0.01, *** < 0.001, *n.s.,* no significance. **b** Mutational signatures of all trunk and non-trunk mutations was inferred by deconstructSigs. Signatures were displayed according to the 96-substitution classification defined by the change class and sequence context. **c** Pie chart showed contributions of mutational signatures to each patient. Signatures were based on the Wellcome Trust Sanger Institute Mutational Signature Framework

Next, we analyzed the mutational spectra of mutations on both trunk and non-trunk to determine the dynamics of mutagenic processes in GBC. The C > T transition was the most dominant change in GBC, which was consistent with a previous study of GBC [7]. The C > A transition and the T > C transition were the second and the third dominant changes in GBC (Fig. 4a). The C > T transition is prevalent in many tumors [16, 17], while the C > A and T > C changes are considered the characteristic signatures of GBC genome [7]. These results demonstrated that these established PDPCs inherited the mutation patterns from the source GBC tissues. The distribution of the six mutation classes in trunk and non-trunk mutations varied from patient to patient. The overall distribution was significantly different (*P* < 0.001, proportion test) between trunk and non-trunk mutations for patients 1279, 4160, 4256, 668 and 902, while no statistical differences were detected for patients 1405 and 1436 (Fig. 4a). In addition to the different distribution in the six mutation classes, differences in the 96 trinucleotide mutational signatures were also observed (Fig. 4b). We next deconstructed contributions of individual mutational signatures to each patient, and identified several dominant signatures in these tumors, including Signature 1 (associated with age), Signature 2 (associated with APOBEC), Signature 4 (associated with smoking), Signatures 6 and 15 (associated with DNA mismatch repair), and Signatures 7 and 17 (Fig. 4c). Moreover, the number of contributed signatures in non-trunk mutations was higher than that in trunk mutations for most of the patients, except patient 1436. Signature 1 was always dominant for trunk mutations across all patients, while signature compositions were quite complex for non-trunk mutations in most patients. For example, signature 6 was dominant in patient 902, signature 15 was dominant in patient 1405, and signatures 1 and 4 were almost equivalent in patient 668.

### ITH of CNVs in GBC

**Fig. 5:**
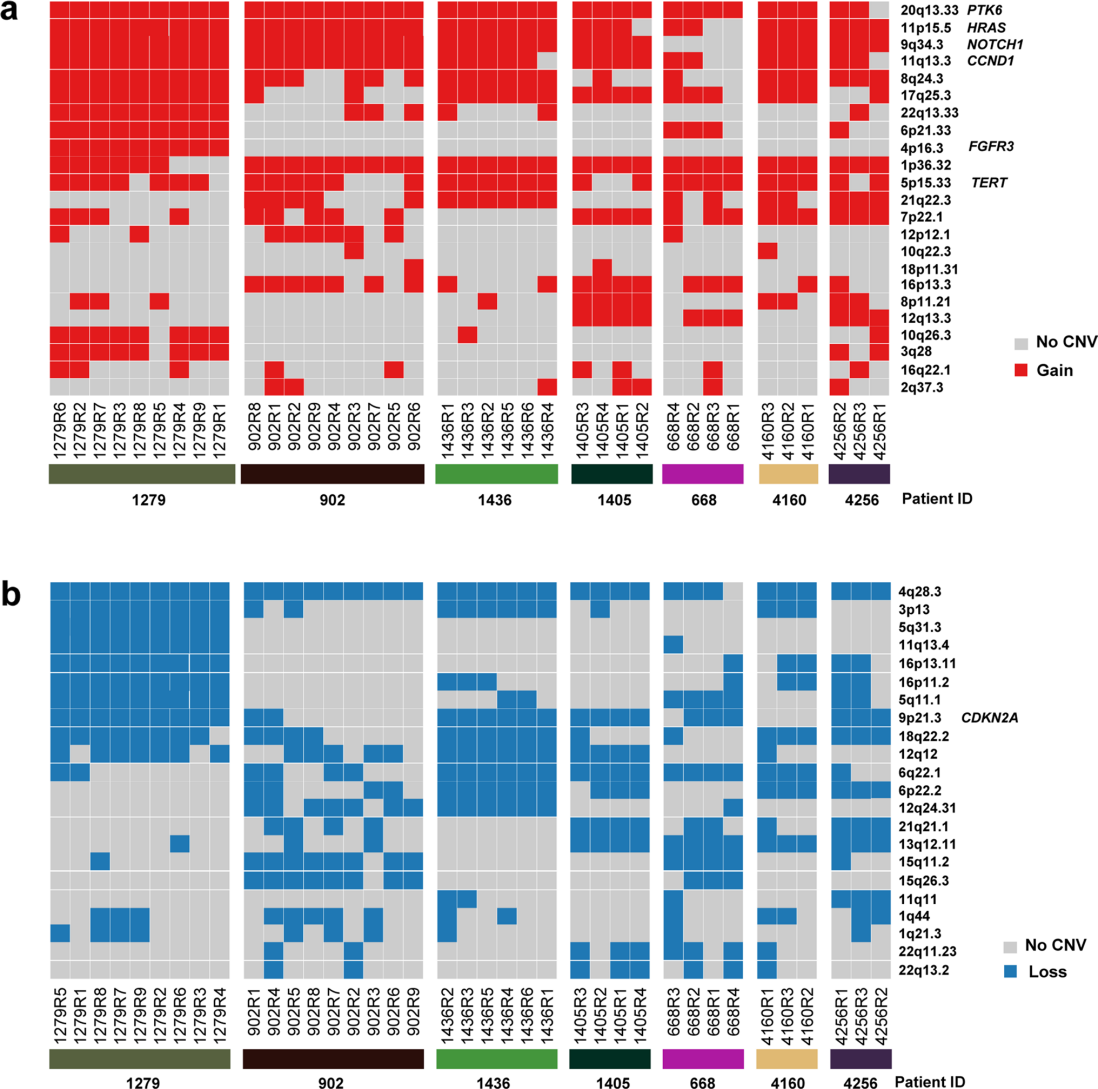
ITH of CNVs in seven GBC patients. **a**, **b** Heat map of cytoband level copy number gain and copy number loss in each sample of the GBC patients. Tumor related genes were shown nearby the corresponding cytobands

We next analyzed the ITH at the CNV level in GBC, focusing on the cytoband level regions. The 23 and 22 CNV peaks were identified as amplifications and deletions in our cohort using GISTIC2.0, respectively (Fig. 5a, b). Notably, six oncogenes were ubiquitously amplified in at least one patient, including *PTK6*, *HRAS*, *NOTCH1*, *CCND1*, *FGFR3*, and *TERT*. *CDKN2A* deletion was ubiquitously identified in 4 patients. ITH varied at the CNV level across different patients. For example, most CNVs (both amplifications and deletions) were ubiquitously identified across different samples of patient 1279, while the CNVs in patient 902 showed much more heterogeneity (especially for deletions) in patient 902.

### ITH of the gene expression profile in GBC

**Fig. 6:**
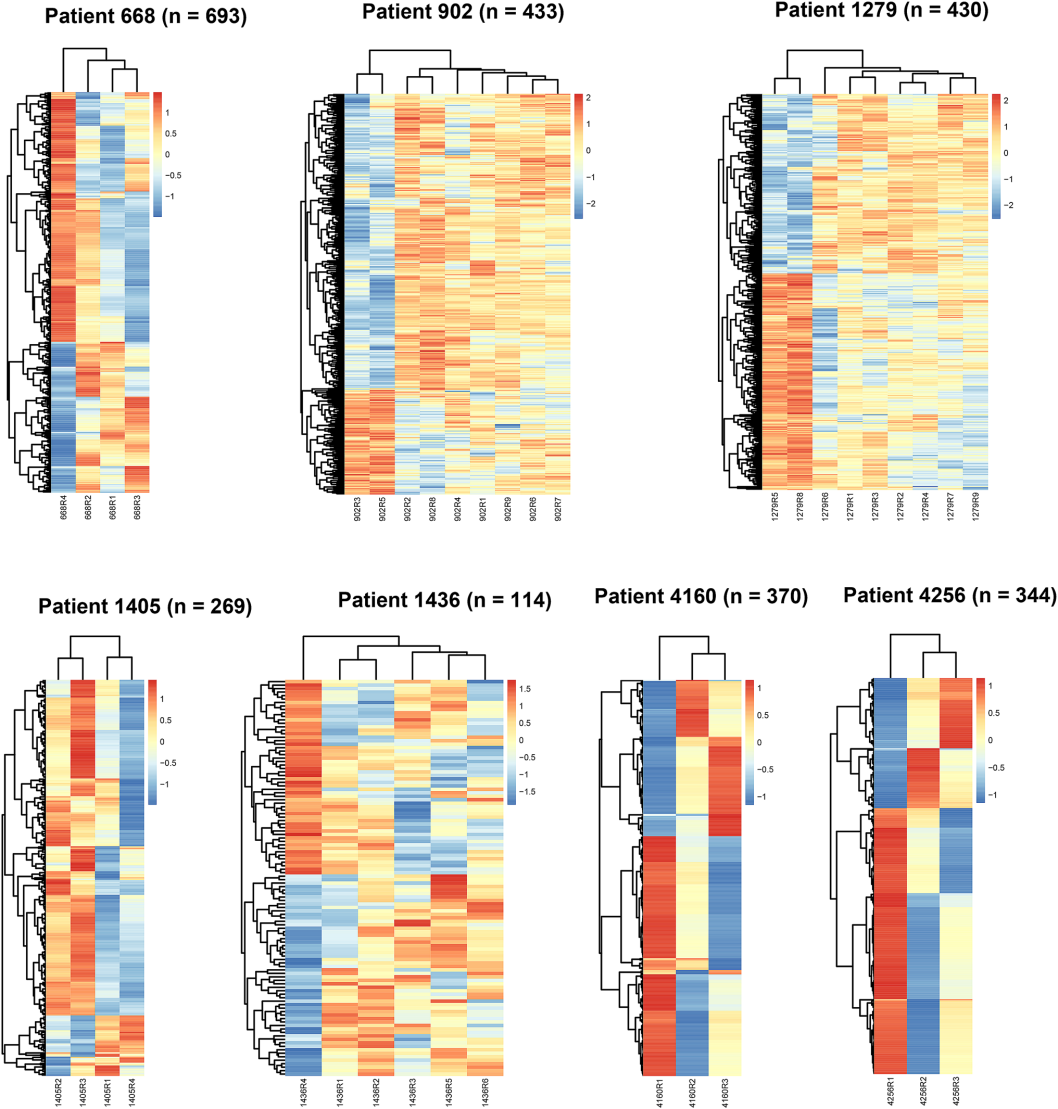
ITH of gene expression profile in seven GBC patients. Unsupervised hierarchical clustering of selected gene expression profiling of seven GBC patients. Rows denoted variably expressed genes across different PDPCs and columns represent samples. Expression was scaled to mean 0 and sd 1 across samples within each patient. The number of genes displayed was shown on the top of each individual panel

Gene expression profiling is a powerful technique widely used in cancer research. To investigate the ITH at the gene expression level in GBC, RNA-seq was performed on an Illumina X Ten instrument for all the 38 PDPCs. A median of 25.7 M read pairs was obtained per sample. To explore the ITH in each GBC patient, candidate DEGs were used for visualization and analysis. A median of 370 DEGs (range, 114–693) was identified per patient according to our method (Fig. 6). Various degrees of ITH were observed in out cohort. There were 114 DEGs identified across samples from patient 1436, which indicated the lowest ITH; while patient 668 exhibited the highest ITH (693 DEGs). Notably, the expression profiles of two PDPCs (R3 and R5) in patient 902 varied from those in the remaining seven samples, which was the same situation as the mutations.

### Heterogeneous expression profile of human leukocyte antigen (HLA) genes

**Fig. 7:**
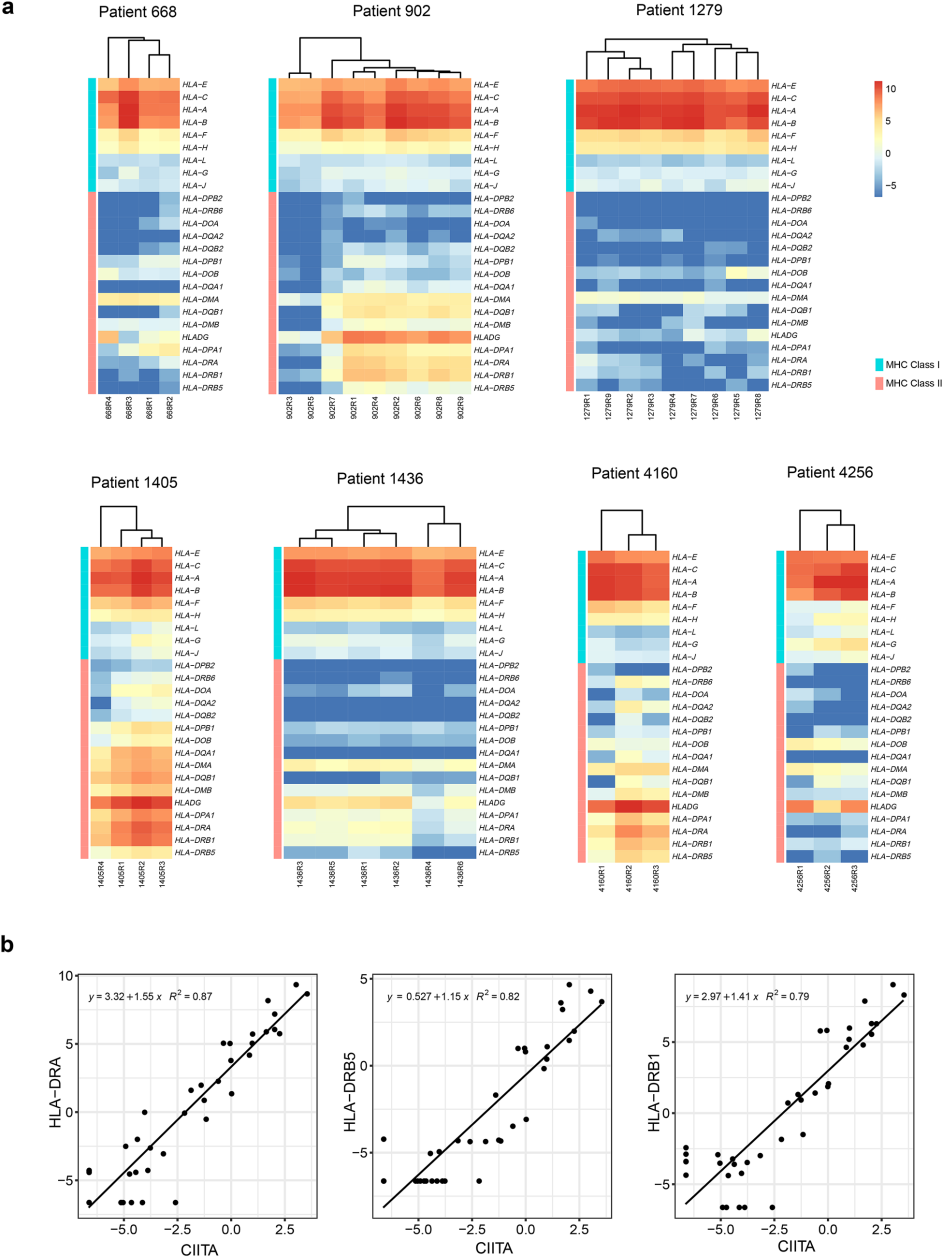
Heterogeneous expression profile of HLA genes. **a** Unsupervised hierarchical clustering of 25 HLA genes including 9 MHC class I and 16 MHC class II genes of seven GBC patients. **b** Scatterplot of three MHC class II genes against *CIITA*. A linear regression formula and Pearson correlation were listed on the top left of each panel

To explore the potential biological relevance underlying these DEGs, we performed a GO enrichment analysis on these DEGs for each patient. It was found these DEGs were involved in cancer-related biological processes, such as cell motility, cell migration and cell proliferation, in several patients (Supplementary Fig. 3). Interestingly, DEGs identified from patient 902 and patient 1436 were enriched in biological processes related to the innate immune response and immune system process. Further investigation revealed that DEGs involved in these processes included several MHC class II genes, such as *HLADG* (*CD74*), *HLA-DPA1*, *HLA-DRA*, *HLA-DRB1*, and *HLA-DRB5*. We then extended to all the HLA genes (including 9 MHC class I and 16 MHC class II genes) to investigate the expression profile across all our patients. Notably, distinct expression patterns were observed in these genes across different patients (Fig. 7a). Most of the MHC class I genes (6 out of 9 genes, except *HLA-G*, *HLA-L*, and *HLA-J*, which were always lowly expressed) were always highly expressed across all patients, while the expression patterns of MHC class II genes were quite complex across different patients. In general, expression of MHC class II genes was much lower than that of MHC class I genes. Several genes (such as *HLA − DPB2*, *HLA − DRB6*, *HLA − DOA*, *HLA − DQA2*, and *HLA − DQB2*) were barely expressed across all samples, while some other genes, such as *HLADG* (*CD74*), *HLA-DPA1*, *HLA-DRA*, *HLA-DRB1*, and *HLA-DRB5*, were found to be expressed in a part of PDPCs in some of the patients. For example, two PDPCs (R3 and R5) from patient 902 did not express these genes, while the remaining PDPCs from this patient had a relatively high expression. Moreover, these genes were ubiquitously expressed across all PDPCs from patient 1405 and patient 4160. In summary, expression of these MHC class II genes varied across different PDPC samples and GBC patients, indicating the transcriptional ITH in GBC.

To investigate which gene may regulate the expression of these MHC class II genes, we next performed a paired association analysis using these MHC class II genes and the other genes in the profiling across all 38 PDPCs. The expression of *CIITA* was most correlated to MHC class II genes (*HLA − DRA*, *HLA − DRB5*, and *HLA − DRB1*, *R*^2^ = 0.87, 0.82 and 0.79, respectively; Fig. 7b), while its expression was not correlated to MHC class I genes (Supplementary Fig. 4).

## Discussion

GBC is an aggressive carcinoma with poor prognosis. Due to relatively low frequency compared to other cancers, the mutation spectrum of this cancer is limited [7, 8]. Rare study assessing ITH of GBC has been reported. To our knowledge, this is the first time to integrate PDPC model, multiregional WES, and RNA-seq to investigate the genomic and transcriptomic ITH of GBC.

Most previous studies have adopt the sampling in different regions of tumor tissue to assess the ITH of various carcinomas [16, 17]. However, low tumor purity of sampling the tumor tissue samples resulted in the impacts on the accuracy of ITH assessment [14]. PDPCs were derived from tumor tissue samples with their high purity and cell population representativeness [43]. Using PDPC models, previous studies identified a median of 60.3% ITH index in ICC [14] and a mean 39.7% ITH index in hepatocellular carcinomas [19]. Several previous studies have reported the PDPCs of GBC [44–49], but none of them explored the ITH of GBC. In this study, we successfully constructed 38 GBC PDPCs, which is the most in all the GBC reports, from the tumor tissue samples of seven patients. Overall, a median 38.3% ITH score of GBC was identified in the PDPC model for the first time.

A limited number of putative driver mutations were identified in each GBC patient, and majority of the mutations were exclusive to patients. *TP53* mutation was ubiquitously truncal across the PDPCs of four out of seven GBC patients. In addition, there were three genes, *KMT2C*, *CDKN2A*, and *ARID1A*, with truncal mutations in at least two GBC patients. Besides that, we found that 12 CCA PDPCs derived from three patients were identified to carry the mutations of recurrently putative driver genes including *TP53*, *KMT2C*, *ERBB2*, and *JAK3* (Supplementary Fig. 5). The mutational results suggested that our GBC and CCA PDPCs recaptured some genomic characteristics of the tumor tissue samples of patients [7–9]. Therefore, in addition to describing ITH features, these PDPCs have the potential to be powerful tools for the studies on drug sensitivity or resistance as well as functional researches.

Transcriptomic analysis has been widely used in cancer studies through a single-sampling approach [50, 51]. However, little is known about the intra-tumoral diversity at the expression level. Herein, we identified transcriptional ITH in GBC. GO enrichment analysis with heterogeneously expressed genes across different samples revealed several biological processes related to tumor development. Notably, the expression patterns of HLA genes were found to be highly diverse from patient to patient. As already known, MHC class II genes are constitutively expressed in antigen-presenting cells (APCs). However, the expression of these genes is undetectable or very low in tumor cells. As previously reported [52], the expression of these genes was highly correlated to *CIITA* in our cohort, which confirmed the regulatory role of *CIITA* in these genes. *CIITA* is solely expressed on professional APCs; however, its expression can be induced by interferon-γ. In a previous study, HLA class II gene expression was identified as a favorable prognostic marker in colorectal carcinoma [53]. Thus, our study provides a clue for the prediction of prognosis in GBC patients.

Although the GBC PDPCs were successfully established and were used to clarify the genomic and transcriptomic ITH, the epigenetics and metabolic ITH were not figured out in this study. In addition, there are several studies on ITH and the drug sensitivity [14, 19, 54]. The relationship between these ITH of GBC and drug sensitivities was not explored. Moreover, we could not explore the association between these ITH and the prognosis of GBC patients due to the lack of therapeutic and prognostic information. Several studies have reported that ITH was considered to be associated with other tumor prognosis [55–57]. Thus, treatment and prognosis information is required to create a comprehensive picture between GBC ITH and prognosis.

## Conclusion

Collectively, we have established and characterized the 38 PDPCs from seven Chinese patients who were diagnosed with gallbladder carcinoma. In addition, we integrated GBC PDPC model, WES, and RNA-seq to reveal the ITH at the gene mutation and transcription levels, which may provide an important molecular foundation for enhanced understanding of tumorigenesis and progression in GBC. These established PDPCs derived from GBC patients can serve as new in vitro models for investigating ITH.

## Supplementary Information

Below is the link to the electronic supplementary material.

Supplementary file1 (PDF 2678 KB)

Supplementary file2 (PDF 521 KB)

## References

[1] Ghidini M, Pizzo C, Botticelli A, Hahne JC, Passalacqua R, Tomasello G (2019). Biliary tract cancer: current challenges and future prospects. Cancer Manag Res.

[2] Baiu I, Visser B (2018). Gallbladder cancer. JAMA.

[3] Hezel AF, Zhu AX (2008). Systemic therapy for biliary tract cancers. Oncologist.

[4] Mohammad YZ, Ghassan KA, Cecilia GE, Shailesh VS, Mahesh G, Bruno N, John P (2019). Evaluation and management of incidental gallbladder cancer. Chin Clin Oncol.

[5] Marcano-Bonilla L, Mohamed EA, Mounajjed T, Roberts LR (2016). Biliary tract cancers: epidemiology, molecular pathogenesis and genetic risk associations. Chin Clin Oncol.

[6] Bridgewater J, Lopes A, Wasan H, Malka D, Jensen L, Okusaka T (2016). Prognostic factors for progression-free and overall survival in advanced biliary tract cancer. Ann Oncol.

[7] Li M, Zhang Z, Li X, Ye J, Wu X, Tan Z (2014). Whole-exome and targeted gene sequencing of gallbladder carcinoma identifies recurrent mutations in the ErbB pathway. Nat Genet.

[8] Nakamura H, Arai Y, Totoki Y, Shirota T, Elzawahry A, Kato M (2015). Genomic spectra of biliary tract cancer. Nat Genet.

[9] Marks EI, Yee NS (2016). Molecular genetics and targeted therapeutics in biliary tract carcinoma. World J Gastroenterol.

[10] Christopher PW, Masashi F, Toru Y, Michele S, Matteo F, Rosa K (2018). Genomic characterization of biliary tract cancers identifies driver genes and predisposing mutations. J Hepatol.

[11] Benjamin AW, Joanne X, Michael RL, Anthony FS, Jimmy JH, Kelsey P (2019). Molecular profiling of biliary cancers reveals distinct molecular alterations and potential therapeutic targets. J Gastrointest Oncol.

[12] Akhilesh P, Eric WS, Steffen D, Harsha G, Leonard DG, Mustafa AB (2020). Integrated genomic analysis reveals mutated ELF3 as a potential gallbladder cancer vaccine candidate. Nat Commun.

[13] Mengdan L, Lihong C, Yiping Q, Fang S, Qi Y, Meiju J (2017). Identification of MAP kinase pathways as therapeutic targets in gallbladder carcinoma using targeted parallel sequencing. Oncotarget.

[14] Dong LQ, Shi Y, Ma LJ, Yang LX, Wang XY, Zhang S (2018). Spatial and temporal clonal evolution of intrahepatic cholangiocarcinoma. J Hepatol.

[15] Pribluda A, de la Cruz CC, Jackson EL (2015). Intratumoral heterogeneity: from diversity comes resistance. Clin Cancer Res.

[16] Gerlinger M, Rowan AJ, Horswell S, Math M, Larkin J, Endesfelder D (2012). Intratumor heterogeneity and branched evolution revealed by multiregion sequencing. N Engl J Med.

[17] Zhang J, Fujimoto J, Zhang J, Wedge DC, Song X, Zhang J (2014). Intratumor heterogeneity in localized lung adenocarcinomas delineated by multiregion sequencing. Science.

[18] Sottoriva A, Spiteri I, Piccirillo SG, Touloumis A, Collins VP, Marioni JC (2013). Intratumor heterogeneity in human glioblastoma reflects cancer evolutionary dynamics. Proc Natl Acad Sci U S A.

[19] Gao Q, Wang ZC, Duan M, Lin YH, Zhou XY, Worthley DL (2017). Cell culture system for analysis of genetic heterogeneity within hepatocellular carcinomas and response to pharmacologic agents. Gastroenterology.

[20] Edge S, Byrd DR, Compton CC (2009). AJCC cancer staging manual.

[21] Tessoulin B, Moreau-Aubry A, Descamps G, Gomez-Bougie P, Maiga S, Gaignard A (2018). Whole-exon sequencing of human myeloma cell lines shows mutations related to myeloma patients at relapse with major hits in the DNA regulation and repair pathways. J Hematol Oncol.

[22] Kohli M, Ho Y, Hillman DW, Van Etten JL, Henzler C, Yang R (2017). Androgen receptor variant AR-V9 Is coexpressed with AR-V7 in prostate cancer metastases and predicts abiraterone resistance. Clin Cancer Res.

[23] Li H, Durbin R (2010). Fast and accurate long-read alignment with burrows-wheeler transform. Bioinformatics.

[24] Cibulskis K, Lawrence MS, Carter SL, Sivachenko A, Jaffe D, Sougnez C (2013). Sensitive detection of somatic point mutations in impure and heterogeneous cancer samples. Nat Biotechnol.

[25] Ye K, Schulz MH, Long Q, Apweiler R, Ning Z (2009). Pindel: a pattern growth approach to detect break points of large deletions and medium sized insertions from paired-end short reads. Bioinformatics.

[26] Wang K, Li M, Hakonarson H (2010). ANNOVAR: functional annotation of genetic variants from high-throughput sequencing data. Nucleic Acids Res.

[27] Genomes Project C, Abecasis GR, Auton A, Brooks LD, DePristo MA, Durbin RM et al (2012). An integrated map of genetic variation from 1,092 human genomes. Nature.

[28] Fu W, O'Connor TD, Jun G, Kang HM, Abecasis G, Leal SM (2013). Analysis of 6,515 exomes reveals the recent origin of most human protein-coding variants. Nature.

[29] Lek M, Karczewski KJ, Minikel EV, Samocha KE, Banks E, Fennell T (2016). Analysis of protein-coding genetic variation in 60,706 humans. Nature.

[30] Forbes SA, Beare D, Boutselakis H, Bamford S, Bindal N, Tate J (2017). COSMIC: somatic cancer genetics at high-resolution. Nucleic Acids Res.

[31] Quinlan AR, Hall IM (2010). BEDTools: a flexible suite of utilities for comparing genomic features. Bioinformatics.

[32] Ong CK, Subimerb C, Pairojkul C, Wongkham S, Cutcutache I, Yu W (2012). Exome sequencing of liver fluke-associated cholangiocarcinoma. Nat Genet.

[33] Farshidfar F, Zheng S, Gingras MC, Newton Y, Shih J, Robertson AG (2017). Integrative genomic analysis of cholangiocarcinoma identifies distinct IDH-mutant molecular profiles. Cell Rep.

[34] Jiao Y, Pawlik TM, Anders RA, Selaru FM, Streppel MM, Lucas DJ (2013). Exome sequencing identifies frequent inactivating mutations in BAP1, ARID1A and PBRM1 in intrahepatic cholangiocarcinomas. Nat Genet.

[35] Rosenthal R, McGranahan N, Herrero J, Taylor BS, Swanton C (2016). DeconstructSigs: delineating mutational processes in single tumors distinguishes DNA repair deficiencies and patterns of carcinoma evolution. Genome Biol.

[36] Alexandrov LB, Nik-Zainal S, Wedge DC, Aparicio SA, Behjati S, Biankin AV (2013). Signatures of mutational processes in human cancer. Nature.

[37] Talevich E, Shain AH, Botton T, Bastian BC (2016). CNVkit: genome-wide copy number detection and visualization from targeted DNA sequencing. PLoS Comput Biol.

[38] Mermel CH, Schumacher SE, Hill B, Meyerson ML, Beroukhim R, Getz G (2011). GISTIC2.0 Facilitates sensitive and confident localization of the targets of focal somatic copy-number alteration in human cancers. Genome Biol.

[39] Dobin A, Davis CA, Schlesinger F, Drenkow J, Zaleski C, Jha S (2013). STAR: ultrafast universal RNA-seq aligner. Bioinformatics.

[40] Wang L, Wang S, Li W (2012). RSeQC: quality control of RNA-seq experiments. Bioinformatics.

[41] Liao Y, Smyth GK, Shi W (2014). featureCounts: an efficient general purpose program for assigning sequence reads to genomic features. Bioinformatics.

[42] Yu G, Wang LG, Han Y, He QY (2012). clusterProfiler: an R package for comparing biological themes among gene clusters. OMICS.

[43] Crystal AS, Shaw AT, Sequist LV, Friboulet L, Niederst MJ, Lockerman EL (2014). Patient-derived models of acquired resistance can identify effective drug combinations for cancer. Science.

[44] Nishida T, Iwasaki H, Johzaki H, Tanaka S, Watanabe R, Kikuchi M (1997). A human gall-bladder signet ring cell carcinoma cell line. Pathol Int.

[45] Liu ZY, Xu GL, Tao HH, Yang YQ, Fan YZ (2017). Establishment and characterization of a novel highly aggressive gallbladder cancer cell line, TJ-GBC2. Cancer Cell Int.

[46] Zhou F, Zhang YH, Sun JH, Yang XM (2019). Characteristics of a novel cell line ZJU-0430 established from human gallbladder carcinoma. Cancer Cell Int.

[47] Shinichi S, Yutaka S, Takuya N, Makoto M, Testuya O, Isaku Y (2012). Establishment and characterization of a new human gallbladder carcinoma cell line. Anticancer Res.

[48] Feng FL, Cheng QB, Yang L, Zhang DD, Ji SL, Zhang QZ (2017). Guidance to rational use of pharmaceuticals in gallbladder sarcomatoid carcinoma using patient-derived cancer cells and whole exome sequencing. Oncotarget.

[49] Patricia G, Carolina B, Lorena R, Jaime AE, Helga W, Javier CI (2020). Functional and genomic characterization of three novel cell lines derived from a metastatic gallbladder cancer tumor. Biol Res.

[50] Yamada A, Yu P, Lin W, Okugawa Y, Boland CR, Goel A (2018). A RNA-Sequencing approach for the identification of novel long non-coding RNA biomarkers in colorectal cancer. Sci Rep.

[51] Reuben A, Gittelman R, Gao J, Zhang J, Yusko EC, Wu CJ (2017). TCR repertoire intratumor heterogeneity in localized lung adenocarcinomas: an association with predicted neoantigen heterogeneity and postsurgical recurrence. Cancer Discov.

[52] Ting JP, Trowsdale J (2002). Genetic control of MHC class II expression. Cell.

[53] Sconocchia G, Eppenberger-Castori S, Zlobec I, Karamitopoulou E, Arriga R, Coppola A (2014). HLA class II antigen expression in colorectal carcinoma tumors as a favorable prognostic marker. Neoplasia.

[54] Bruna A, Rueda OM, Greenwood W, Batra AS, Callari M, Batra RN (2016). A biobank of breast cancer explants with preserved intra-tumor heterogeneity to screen anticancer compounds. Cell.

[55] Qazi MA, Vora P, Venugopal C, Sidhu SS, Moffat J, Swanton C (2017). Intratumoral heterogeneity: pathways to treatment resistance and relapse in human glioblastoma. Ann Oncol.

[56] Morris LG, Riaz N, Desrichard A, Senbabaoglu Y, Hakimi AA, Makarov V (2016). Pan-cancer analysis of intratumor heterogeneity as a prognostic determinant of survival. Oncotarget.

[57] Hou Y, Nitta H, Wei L, Banks PM, Portier B, Parwani AV (2017). HER2 intratumoral heterogeneity is independently associated with incomplete response to anti-HER2 neoadjuvant chemotherapy in HER2-positive breast carcinoma. Breast Cancer Res Treat.

