# Supplementary material for: Establishment and characterization of 38 novel patient-derived primary cancer cell lines using multi-region sampling revealing intra-tumor heterogeneity of gallbladder carcinoma
Source: Hum Cell. 2021 Apr 4;34(3):918–31. doi: 10.1007/s13577-021-00492-5 (PMC8057967; doi:10.1007/s13577-021-00492-5)
Supplement: Supplementary file 1 — Supplementary file1 (PDF 2678 KB) [file 13577_2021_492_MOESM1_ESM.pdf]

Supplementary Figures

Establishment and characterization of 38 novel patient-derived primary cancer cell lines using multi-region sampling revealing intra-tumor heterogeneity of gallbladder carcinoma

Feiling Feng[1], Qingbao Cheng[1], Bin Li[2], Chen Liu[1], Huizhen Wang[2], Bin Li[1], Xiaoya Xu[2], Yong Yu[1], Zishuo Chen[2], Xiaobing Wu[1], Hua Dong[2], Kaijian Chu[1], Zhenghua Xie[2], Qingxiang Gao[1], Lei Xiong[2], Fugen Li[2], Bin Yi[1], Dadong Zhang[2], Xiaoqing Jiang[1]

[1]Department of Biliary I, Shanghai Eastern Hepatobiliary Surgery Hospital, Navy Military Medical University, Shanghai 200438, China

[2]3D Medicines Inc., Shanghai 201114, China

Correspondence: Xiaoqing Jiang, e-mail: xqjiang_dandaoyike@163.com; Dadong Zhang, e-mail: dadong.zhang@3dmedcare.com.

Supplementary Figure 1. The morphology of 38 PDPCs derived from 7 GBC patients

Supplementary Figure 2. Sample clustering with SNPs

Supplementary Figure 3. **Gene Ontology (GO) enrichment analysis with DEGs.**

Supplementary Figure 4. **Scatterplot of three MHC class I genes against CIITA.**

Supplementary Figure 5. **ITH of mutations in three patients with cholangiocarcinoma.**

Supplementary Figure 1

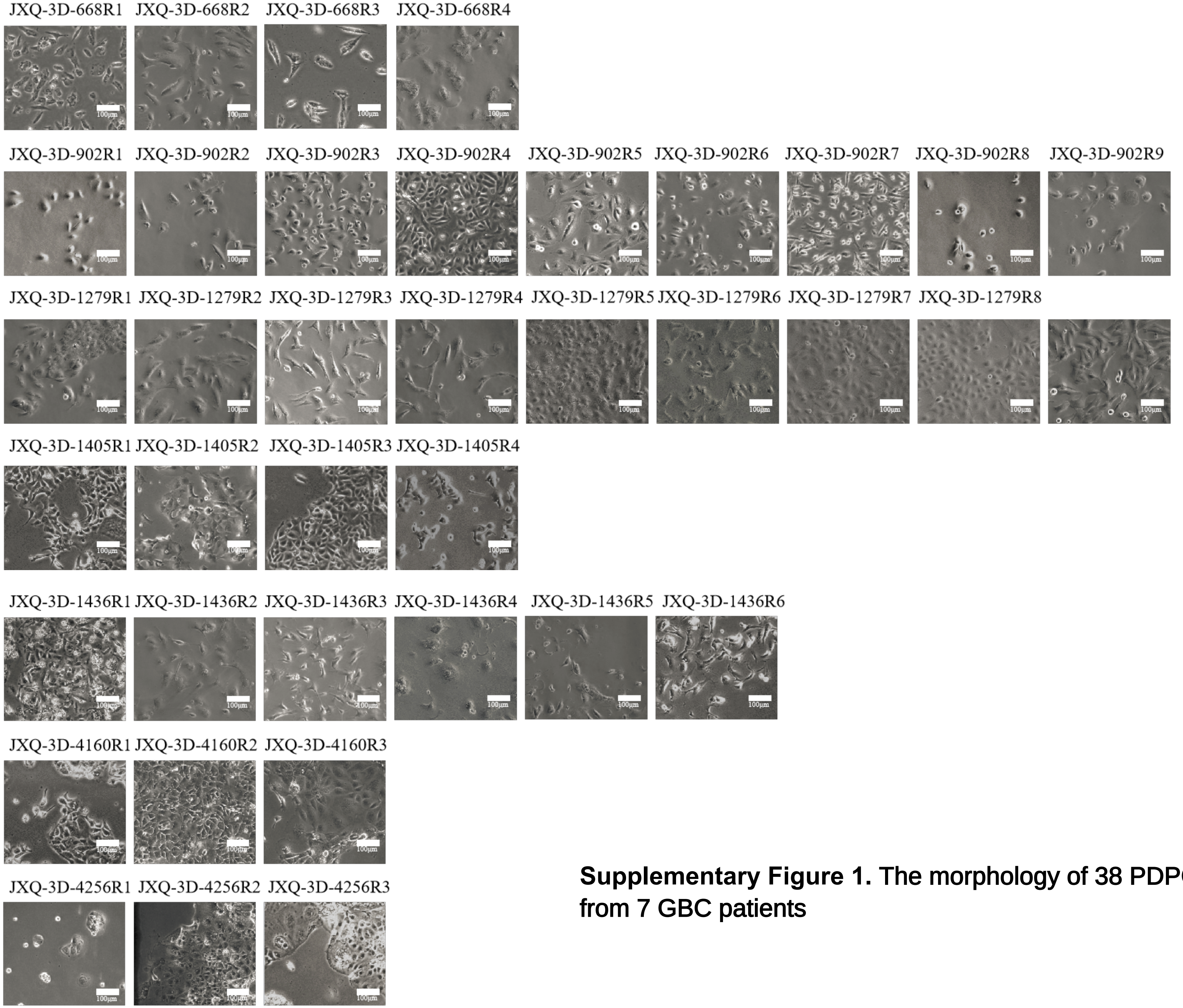

Supplementary Figure 1. The morphology of 38 PDPCs derived from 7 GBC patients

Supplementary Figure 2

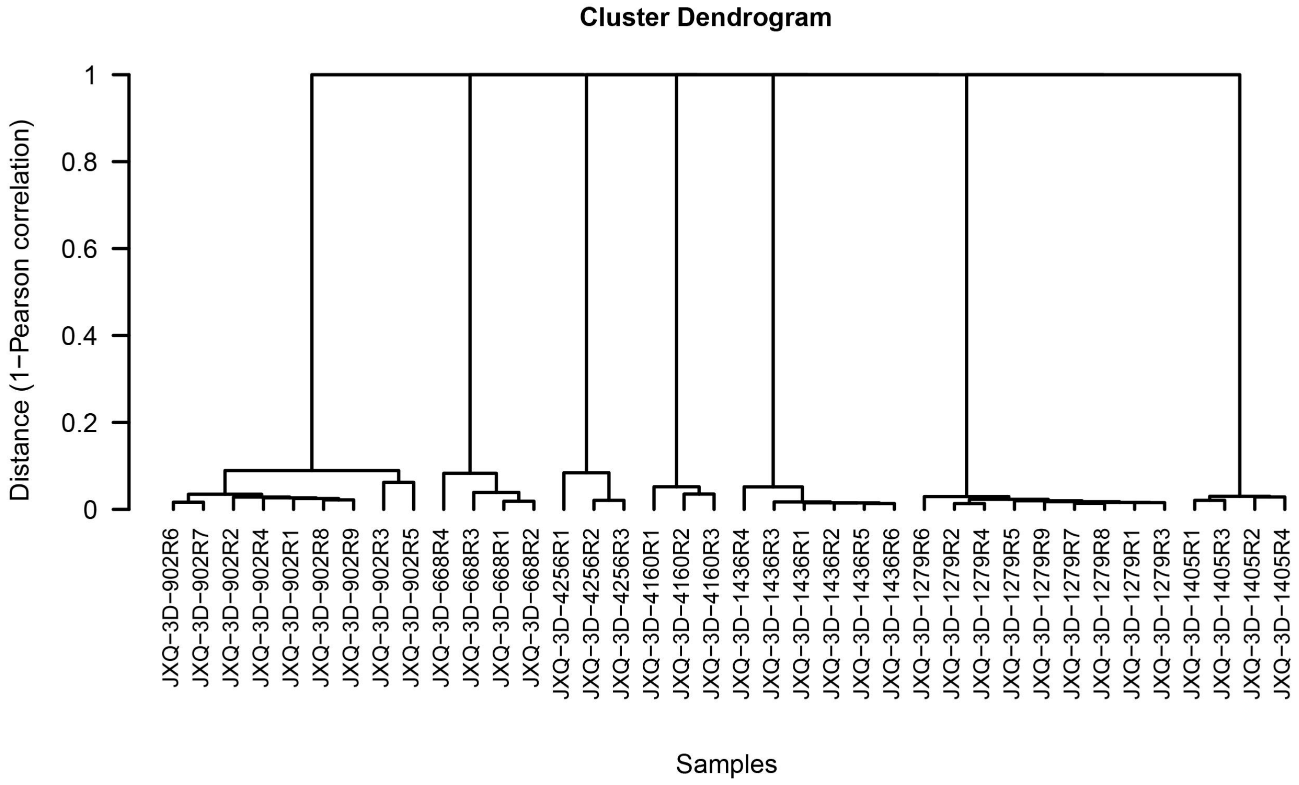

Supplementary Figure 2 . Sample clustering with SNPs

Samples were clustered based on the presence/absence of given SNPs. Samples from the same patient were clustered together which confirmed the origin of these PDC samples.

Supplementary Figure 3

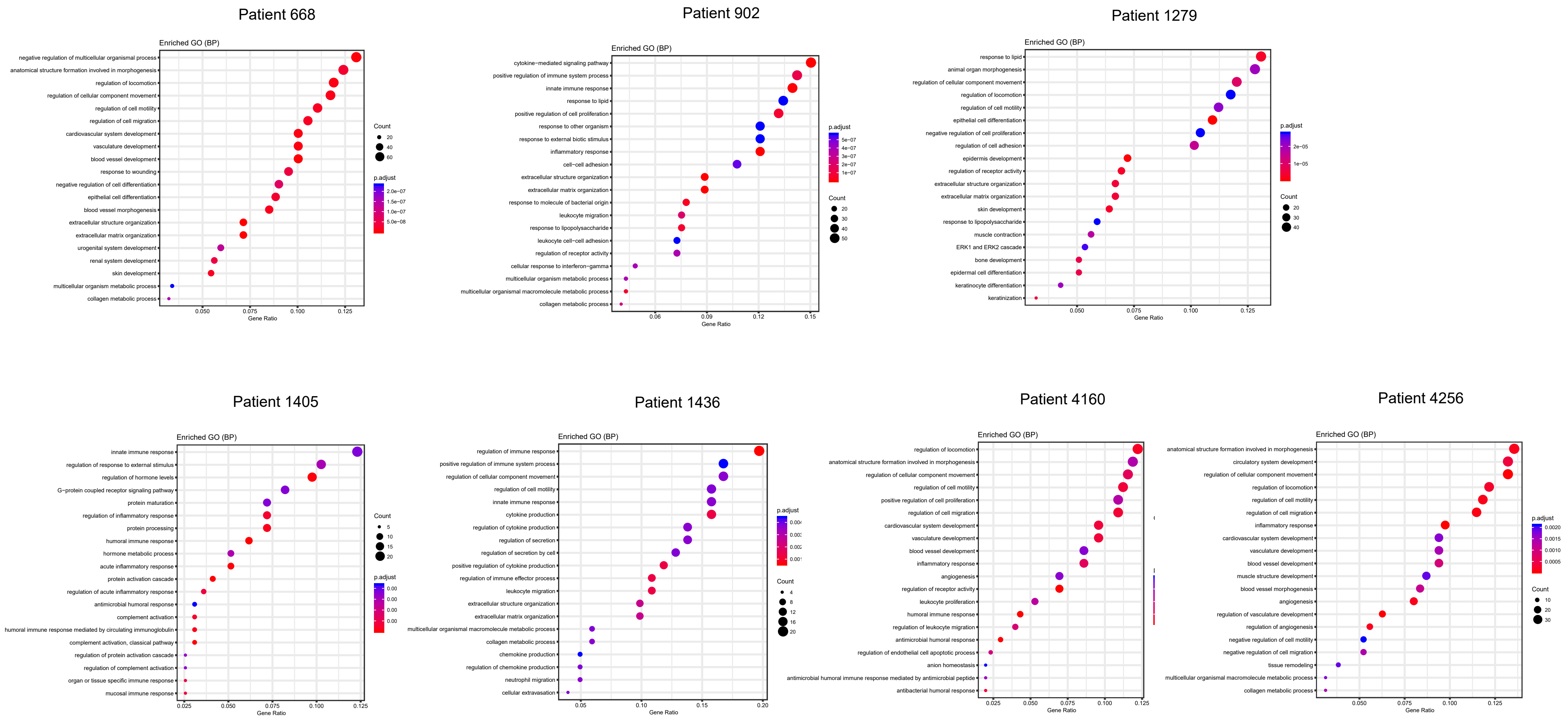

Supplementary Figure 3. Gene Ontology (GO) enrichment analysis with DEGs.
Each panel showed the enriched GO terms of biological processes for each GBC patient.

Supplementary Figure 4

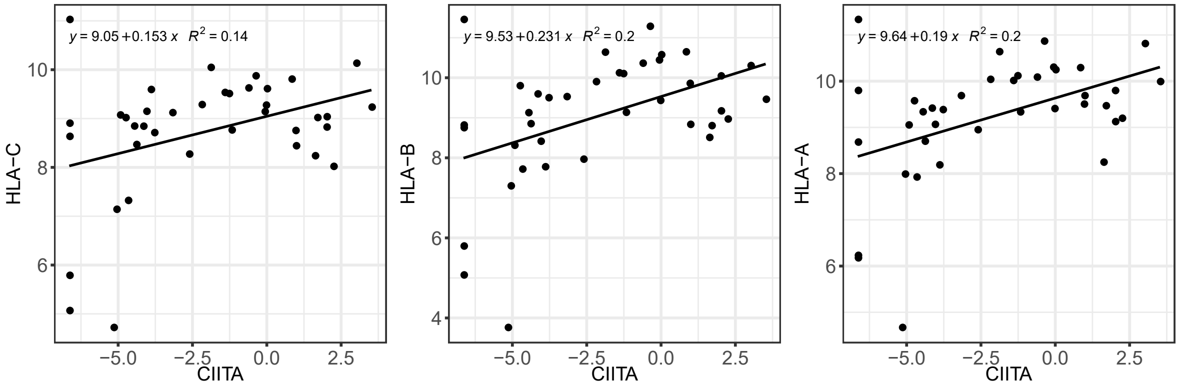

Supplementary Figure 4. Scatterplot of three MHC class I genes against CIITA.
A linear regression formula and pearson correlation were listed on the top left of each panel.

Supplementary Figure 5

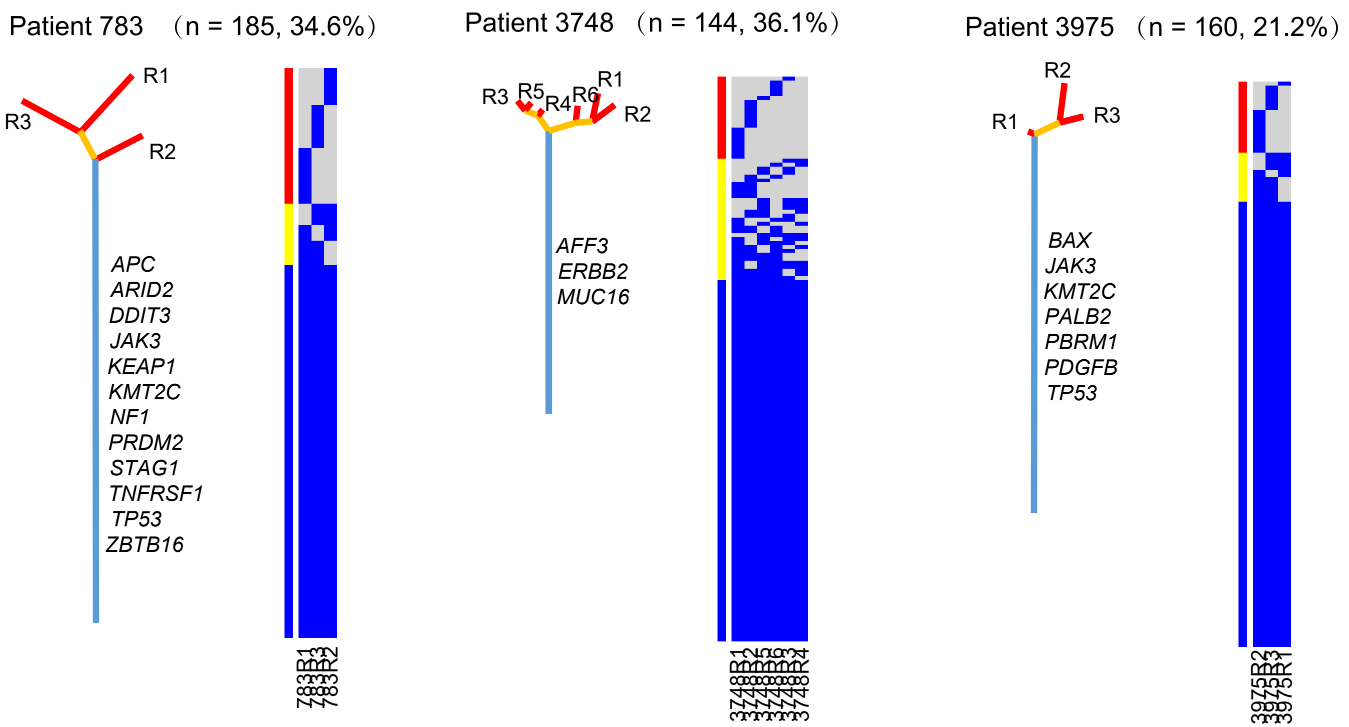

Supplementary Figure 5. ITH of mutations in three patients with cholangiocarcinoma. Phylogenetic trees were generated from somatic mutations using the parsimony ratchet method, and the branch lengths were scaled in proportion to the number of variants (see Methods). Heat map nearby each tree showed the occurrence (presence in blue and absence in grey) of each mutation in each PDC. Genes with putative driver mutations were displayed beside the trunk branch of each individual. The number of mutations and the ITH score were listed on the top of each individual panel. Blue: trunk mutations, yellow: shared mutations, red: private mutations.
