# Supplementary material for: Establishment and characterization of 38 novel patient-derived primary cancer cell lines using multi-region sampling revealing intra-tumor heterogeneity of gallbladder carcinoma
Source: Hum Cell. 2021 Apr 4;34(3):918–31. doi: 10.1007/s13577-021-00492-5 (PMC8057967; doi:10.1007/s13577-021-00492-5)
Supplement: Supplementary file 2 — Supplementary file2 (PDF 521 KB) [file 13577_2021_492_MOESM2_ESM.pdf]

Supplementary Tables

Establishment and characterization of 38 novel patient-derived primary cancer cell lines using multi-region sampling revealing intra-tumor heterogeneity of gallbladder carcinoma

Feiling Feng[1], Qingbao Cheng[1], Bin Li[2], Chen Liu[1], Huizhen Wang[2], Bin Li[1], Xiaoya Xu[2], Yong Yu[1], Zishuo Chen[2], Xiaobing Wu[1], Hua Dong[2], Kaijian Chu[1], Zhenghua Xie[2], Qingxiang Gao[1], Lei Xiong[2], Fugen Li[2], Bin Yi[1], Dadong Zhang[2], Xiaoqing Jiang[1]

[1]Department of Biliary I, Shanghai Eastern Hepatobiliary Surgery Hospital, Navy Military Medical University, Shanghai 200438, China

[2]3D Medicines Inc., Shanghai 201114, China

Correspondence: Xiaoqing Jiang, e-mail: xqjiang_dandaoyike@163.com; Dadong Zhang, e-mail: dadong.zhang@3dmedcare.com.

Supplementary Table 1. Karyotype results of 38 GBC PDCs

Supplementary Table 2. STR results of 38 GBC PDCs

Supplementary Table 1. Karyotype results of 38 GBC PDCs

PDC_ID	Identification results (karyotype)
JXQ-3D-668R1	59； 60； 60； 60； 60； 61； 61； 61； 61； 62； 62； 62； 62； 62； 63； 63； 63； 63； 63； 63
JXQ-3D-668R2	51； 54； 56； 58； 59； 59； 60； 60； 60； 60； 61； 61； 61； 61； 61； 62； 62； 63； 63； 63
JXQ-3D-668R3	46； 46； 47； 48； 49； 50； 50； 51； 51； 51； 52； 54； 56； 56； 58； 58； 59； 60； 71； 80
JXQ-3D-668R4	51； 52； 53； 56； 56； 59； 59； 59； 60； 61； 61； 62； 65； 69； 70； 73； 100； 111； 113
JXQ-3D-1279R1	60； 60； 61； 61； 61； 62； 62； 62； 62； 62； 62； 63； 64； 64； 64； 65； 65； 66； 66； 68
JXQ-3D-1279R2	62； 62； 62； 63； 63； 63； 63； 64； 64； 64； 65； 65； 66； 66； 66； 66； 67； 68； 68； 69
JXQ-3D-1279R3	59； 59； 60； 61； 62； 63； 63； 63； 64； 64； 64； 65； 65； 65； 65； 66； 66； 67； 67； 67
JXQ-3D-1279R4	58； 60； 62； 64； 65； 65； 67； 67； 68； 69； 71； 72； 73； 73； 74； 74； 93； 104； 128； 129
JXQ-3D-1279R5	48； 56； 58； 59； 59； 63； 65； 65； 66； 66； 66； 66； 66； 67； 68； 104； 105； 110； 112； 118
JXQ-3D-1279R6	58； 59； 59； 59； 60； 61； 61； 61； 62； 62； 62； 63； 64； 64； 64； 64； 65； 67； 68； 69
JXQ-3D-1279R7	58； 61； 62； 62； 62； 62； 60； 64； 64； 65； 65； 65； 65； 66； 66； 66； 66； 67； 67； 70
JXQ-3D-1279R8	57； 59； 61； 62； 63； 63； 63； 64； 64； 65； 66； 67； 67； 68； 68； 68； 68； 68； 71； 72
JXQ-3D-1279R9	59； 62； 63； 64； 65； 65； 67； 68； 74； 75； 76； 77； 79； 79； 86； 88； 94； 103； 107； 110
JXQ-3D-1405R1	59； 60； 62； 64； 64； 64； 66； 66； 67； 67； 67； 68； 68； 68； 69； 69； 69； 70； 70； 73
JXQ-3D-1405R2	56； 56； 57； 60； 60； 61； 62； 62； 63； 63； 63； 63； 64； 64； 66； 66； 67； 67； 68； 68
JXQ-3D-1405R3	61； 61； 62； 62； 62； 63； 63； 63； 63； 64； 64； 64； 66； 66； 66； 66； 66； 66； 66； 70
JXQ-3D-1405R4	55； 55； 55； 55； 56； 56； 58； 58； 58； 59； 59； 59； 59； 59； 59； 59； 60； 60； 60； 63
JXQ-3D-1436R1	68； 69； 70； 70； 71； 71； 71； 71； 72； 73； 73； 73； 73； 74； 74； 74； 75； 76； 76； 77
JXQ-3D-1436R2	58； 58； 59； 60； 63； 64； 64； 64； 64； 65； 65； 66； 67； 67； 68； 68； 69； 69； 70； 71
JXQ-3D-1436R3	52； 54； 54； 55； 58； 58； 62； 62； 62； 64； 66； 70； 70； 70； 70； 70； 74； 76； 78； 78
JXQ-3D-1436R4	60； 60； 61； 61； 62； 62； 63； 63； 63； 64； 65； 65； 67； 67； 69； 70； 71； 72； 73； 75
JXQ-3D-1436R5	64； 64； 64； 66； 66； 66； 66； 67； 67； 67； 68； 68； 68； 69； 69； 71； 73； 73； 73； 73
JXQ-3D-1436R6	51； 54； 55； 58； 59； 59； 59； 60； 61； 62； 62； 62； 63； 63； 64； 64； 65； 69； 71； 73
JXQ-3D-902R1	66； 67； 69； 69； 70； 71； 71； 71； 72； 72； 72； 72； 72； 73； 73； 75； 75； 76； 77； 77
JXQ-3D-902R2	60； 62； 62； 64； 64； 64； 65； 65； 66； 66； 67； 67； 68； 68； 68； 68； 68； 68； 68； 69
JXQ-3D-902R3	54； 56； 57； 58； 59； 60； 61； 61； 62； 62； 63； 64； 66； 66； 68； 73； 86； 90； 90； 96
JXQ-3D-902R4	70； 71； 71； 71； 71； 72； 72； 73； 74； 75； 75； 76； 76； 78； 80； 80； 80； 80； 81； 83
JXQ-3D-902R5	62； 65； 65； 69； 71； 72； 73； 75； 77； 77； 78； 80； 80； 81； 81； 81； 83； 84； 84； 85
JXQ-3D-902R6	54； 55； 56； 56； 58； 58； 60； 60； 61； 62； 62； 63； 63； 63； 64； 65； 65； 65； 66； 67
JXQ-3D-902R7	59； 60； 61； 62； 63； 63； 63； 65； 65； 65； 66； 67； 67； 68； 68； 68； 70； 70； 71； 71
JXQ-3D-902R8	59； 60； 60； 60； 61； 61； 61； 61； 61； 61； 62； 62； 63； 64； 65； 65； 66； 68； 68； 68
JXQ-3D-902R9	62； 63； 64； 65； 66； 66； 66； 67； 67； 69； 70； 70； 71； 71； 71； 71； 72； 72； 72； 74
JXQ-3D-4160R1	53； 56； 57； 59； 64； 64； 65； 65； 66； 67； 67； 68； 69； 71； 71； 72； 72； 72； 73； 73
JXQ-3D-4160R2	65； 67； 69； 71； 71； 71； 71； 73； 73； 73； 74； 74； 75； 76； 76； 76； 78； 78； 79； 79
JXQ-3D-4160R3	61； 66； 67； 69； 71； 71； 72； 73； 73； 75； 75； 75； 77； 78； 78； 80； 80； 80； 86； 86
JXQ-3D-4256R1	46； 46； 46； 47； 47； 47； 48； 49； 50； 51； 51； 51； 52； 52； 53； 54； 54； 54； 56； 56
JXQ-3D-4256R2	46； 48； 48； 48； 49； 51； 51； 51； 52； 54； 55； 56； 56； 56； 57； 58； 58； 59； 59； 63
JXQ-3D-4256R3	47； 49； 49； 50； 54； 54； 54； 56； 56； 56； 61； 63； 65； 70； 72； 84； 88； 90； 97； 100

Supplementary Table 2. STR results of 38 GBC PDCs

Marker	JXQ-3D-668R1		JXQ-3D-668R2		JXQ-3D-668R3		JXQ-3D-668R4	
	Allele1	Allele2	Allele1	Allele2	Allele1	Allele2	Allele1	Allele2
D5S818	9	11	9	11	9	9	9	11
D13S317	8	9	8	9	8	9	8	9
D7S820	11	12	11	12	11	12	11	12
D16S539	11	12	11	12	11	12	11	12
VWA	14	17	14	17	14	17	14	17
TH01	6	9	6	9	6	9	6	9
AMEL	X	X	X	X	X	X	X	Y
TPOX	9	11	9	11	9	11	8	9
CSF1PO	12	12	12	12	12	12	12	12
D12S391	19	26	19	26	19	26	19	26
FGA	24.2	24.2	22	24.2	24.2	24.2	22	24.2
D2S1338	19	26	19	26	19	26	19	26
D21S11	31.2	31.2	31.2	31.2	31.2	31.2	31.2	31.2
D18S51	14	16	14	16	14	16	14	16
D8S1179	12	12	12	12	12	12	12	13
D3S1358	15	17	15	17	15	17	15	17
D6S1043	14	14	14	14	14	14	14	14
PENTAE	15	15	15	15	15	15	15	15
D19S433	13	15.2	13	15.2	13	15.2	13	15.2
PENTAD	9	9	9	9	9	9	9	9

Marker	JXQ-3D-1279R1		JXQ-3D-1279R2		JXQ-3D-1279R3		JXQ-3D-1279R4	
	Allele1	Allele2	Allele1	Allele2	Allele1	Allele2	Allele1	Allele2
D5S818	11	11	11	11	11	11	11	11
D13S317	11	12	11	12	11	12	11	12
D7S820	10	12	10	12	10	12	10	12
D16S539	10	11	10	11	10	11	10	11
VWA	19	19	19	19	19	19	19	19
TH01	7	7	7	7	7	7	7	7
AMEL	X	Y	X	Y	X	X	X	Y
TPOX	8	11	8	11	8	11	8	11
CSF1PO	9	9	9	9	9	9	9	9
D12S391	17	17	17	17	17	17	17	17
FGA	19	19	19	19	19	19	19	19
D2S1338	16	19	16	19	16	19	16	19
D21S11	29	30	29	32.2	29	30	29	32.2
D18S51	14	16	14	16	14	16	14	16
D8S1179	10	15	10	15	10	15	10	15
D3S1358	15	15	15	15	15	15	15	15
D6S1043	12	14	12	14	12	14	12	14
PENTAE	5	11	5	11	5	11	5	11
D19S433	13.2	13.2	13.2	13.2	13.2	13.2	13.2	13.2
PENTAD	9	9	9	9	9	9	9	9

Marker	JXQ-3D-1405R4		JXQ-3D-1436R1		JXQ-3D-1436R2		JXQ-3D-1436R3	
	Allele1	Allele2	Allele1	Allele2	Allele1	Allele2	Allele1	Allele2
D5S818	11	12	11	12	11	12	11	12
D13S317	11	12	8	10	8	10	8	10
D7S820	8	12	12	12	12	12	12	12
D16S539	9	10	9	10	9	10	9	10
VWA	14	18	16	18	16	18	16	18
TH01	9	9	9	9	9	9	9	9
AMEL	X	X	X	Y	X	Y	X	Y
TPOX	8	11	8	11	8	11	8	11
CSF1PO	9	13	10	10	10	10	10	10
D12S391	18	18	15	17	15	17	15	17
FGA	21	25	23	24	23	24	23	24
D2S1338	22	23	17	25	17	25	17	25
D21S11	30	30	29	31	29	31	29	31
D18S51	12	17	14	14	14	14	14	14
D8S1179	10	13	13	13	13	13	13	13
D3S1358	15	17	15	16	15	16	15	16
D6S1043	19	19	17	20	17	20	17	20
PENTAE	17	19	5	16	5	16	5	16
D19S433	12	13	13	15	13	15	13	15
PENTAD	9	9	10	11	10	11	10	11

Marker	JXQ-3D-1436R4		JXQ-3D-1436R5		JXQ-3D-1436R6		JXQ-3D-902R1	
	Allele1	Allele2	Allele1	Allele2	Allele1	Allele2	Allele1	Allele2
D5S818	11	12	11	12	11	12	11	12
D13S317	8	10	8	10	8	10	9	11
D7S820	12	12	12	12	12	12	8	12
D16S539	9	10	9	10	9	10	10	11
VWA	16	18	16	18	16	18	16	19
TH01	9	9	9	9	9	9	9	9
AMEL	X	Y	X	Y	X	Y	X	X
TPOX	8	11	8	11	8	11	8	12
CSF1PO	10	10	10	10	10	10	12	12
D12S391	15	17	15	17	15	17	20	20
FGA	23	24	23	24	23	24	25	25
D2S1338	17	25	17	25	17	25	19	23
D21S11	29	31	29	31	29	31	31	32
D18S51	14	14	14	14	14	14	17	19
D8S1179	13	13	13	13	13	13	13	14
D3S1358	15	16	15	16	15	16	15	15
D6S1043	17	20	17	20	17	20	11	11
PENTAE	5	16	5	16	5	16	12	12
D19S433	13	15	13	15	13	15	15.2	15.2
PENTAD	10	11	10	11	10	11	8	13

Marker	JXQ-3D-4160R1		JXQ-3D-4160R2		JXQ-3D-4160R3	
	Allele1	Allele2	Allele1	Allele2	Allele1	Allele2
D5S818	10	12	11	12	11	12
D13S317	8	8	8	11	8	8
D7S820	11	11	11	11	11	11
D16S539	10	13	10	11	10	13
VWA	14	16	14	16	14	16
TH01	9	9	9	9	9	9
AMEL	X	X	X	X	X	X
TPOX	8	11	8	11	8	11
CSF1PO	10	13	10	13	10	13
D12S391	19	19	19	21	19	19
FGA	22	23	22	23	22	23
D2S1338	20	23	20	23	20	23
D21S11	28	33.2	28	33.2	28	33.2
D18S51	19	19	11	15	19	19
D8S1179	13	16	13	16	13	16
D3S1358	15	18	15	18	15	18
D6S1043	13	20	13	18	13	20
PENTAE	11	15	11	15	11	15
D19S433	14	15.2	13	14	14	15.2
PENTAD	10	13	10	13	10	13

Marker	JXQ-3D-4525R1		JXQ-3D-4525R2		JXQ-3D-4160R3	
	Allele1	Allele2	Allele1	Allele2	Allele1	Allele2
D5S818	11	11	11	11	11	12
D13S317	9	9	9	9	8	8
D7S820	8	11	8	11	11	11
D16S539	10	10	10	10	10	13
VWA	15	18	15	18	14	16
TH01	7	7	7	7	9	9
AMEL	X	X	X	X	X	X
TPOX	8	8	8	8	8	11
CSF1PO	10	10	10	10	10	13
D12S391	18	18	18	18	19	19
FGA	20	20	20	20	22	23
D2S1338	17	17	17	18	20	23
D21S11	31.2	31.2	31.2	31.2	28	33.2
D18S51	20	20	20	20	19	19
D8S1179	13	15	13	13	13	16
D3S1358	15	15	15	15	15	18
D6S1043	18	18	14	18	13	20
PENTAE	11	19	11	19	11	15
D19S433	14.2	14.2	14.2	14.2	14	15.2
PENTAD	9	11	9	11	10	13

Marker	JXQ-3D-1279R5		JXQ-3D-1279R6		JXQ-3D-1279R7		JXQ-3D-1279R8	
	Allele1	Allele2	Allele1	Allele2	Allele1	Allele2	Allele1	Allele2
D5S818	11	11	11	11	11	11	11	11
D13S317	11	12	11	12	11	12	11	12
D7S820	10	12	10	12	10	12	10	12
D16S539	10	11	10	11	10	11	10	11
VWA	19	19	19	19	19	19	19	19
TH01	7	7	7	7	7	7	7	7
AMEL	X	X	X	Y	X	X	X	Y
TPOX	8	11	8	11	8	11	8	11
CSF1PO	9	9	9	9	9	9	9	9
D12S391	17	17	17	17	17	17	17	17
FGA	19	19	19	19	19	19	19	19
D2S1338	16	19	16	19	16	19	16	19
D21S11	29	30	29	32.2	29	30	29	30
D18S51	14	16	14	14	14	16	14	16
D8S1179	10	15	10	15	10	15	10	15
D3S1358	15	15	15	15	15	15	15	15
D6S1043	12	14	12	14	12	14	12	14
PENTAE	5	11	5	11	5	11	5	11
D19S433	13.2	13.2	13.2	13.2	13.2	13.2	13.2	13.2
PENTAD	9	9	9	9	9	9	9	9

Marker	JXQ-3D-1279R9		JXQ-3D-1405R1		JXQ-3D-1405R2		JXQ-3D-1405R3	
	Allele1	Allele2	Allele1	Allele2	Allele1	Allele2	Allele1	Allele2
D5S818	11	11	11	12	11	12	11	12
D13S317	11	12	11	12	11	12	11	12
D7S820	10	12	8	9	8	12	8	12
D16S539	10	11	9	10	9	10	9	10
VWA	19	19	14	18	14	18	14	18
TH01	7	7	8	9	9	9	9	9
AMEL	X	Y	X	Y	X	X	X	Y
TPOX	8	11	8	11	8	11	8	11
CSF1PO	9	9	9	14	9	13	9	13
D12S391	17	17	18	18	18	18	18	18
FGA	19	19	21	25	21	25	25	25
D2S1338	16	19	22	23	22	23	22	23
D21S11	29	30	30	30	30	30	30	30
D18S51	14	16	12	17	12	17	12	17
D8S1179	10	15	10	13	10	13	10	13
D3S1358	15	15	15	17	15	17	15	17
D6S1043	12	14	19	19	19	19	19	19
PENTAE	5	11	17	18.4	17	19	17	19
D19S433	13.2	13.2	12	13	12	13	12	13
PENTAD	9	9	9	9	9	9	9	9

Marker	JXQ-3D-902R2		JXQ-3D-902R3		JXQ-3D-902R4		JXQ-3D-902R5	
	Allele1	Allele2	Allele1	Allele2	Allele1	Allele2	Allele1	Allele2
D5S818	11	12	11	12	11	12	11	12
D13S317	9	11	9	11	9	11	11	11
D7S820	8	12	8	12	8	12	8	12
D16S539	10	11	10	11	10	11	10	11
VWA	19	19	19	19	19	19	19	19
TH01	9	9	9	9	9	9	9	9
AMEL	X	X	X	X	X	X	X	X
TPOX	8	12	8	12	8	12	8	12
CSF1PO	12	12	12	12	12	12	12	12
D12S391	20	20	20	20	20	20	20	20
FGA	25	25	25	25	25	25	25	25
D2S1338	19	23	19	23	19	23	19	19
D21S11	31	32	31	32	31	32	31	32
D18S51	17	19	17	19	17	19	17	19
D8S1179	13	14	13	14	13	13	13	14
D3S1358	15	15	15	15	15	15	15	15
D6S1043	11	11	11	11	11	11	11	11
PENTAE	12	12	12	12	12	12	12	13
D19S433	15.2	15.2	15.2	15.2	15.2	15.2	15.2	15.2
PENTAD	8	13	8	13	8	13	8	13

Marker	JXQ-3D-902R6		JXQ-3D-902R7		JXQ-3D-902R8		JXQ-3D-902R9	
	Allele1	Allele2	Allele1	Allele2	Allele1	Allele2	Allele1	Allele2
D5S818	11	12	11	12	11	12	11	12
D13S317	9	11	9	11	9	11	9	11
D7S820	8	12	8	12	8	12	8	12
D16S539	10	11	10	11	10	11	10	11
VWA	19	19	19	19	19	19	19	19
TH01	9	9	9	9	9	9	9	9
AMEL	X	X	X	X	X	X	X	X
TPOX	8	12	8	12	8	12	8	12
CSF1PO	12	12	12	12	12	12	12	12
D12S391	20	20	20	20	20	20	20	20
FGA	25	25	25	25	25	25	25	25
D2S1338	19	23	19	23	19	23	19	23
D21S11	31	32	31	32	31	32	31	32
D18S51	17	19	17	19	17	19	17	19
D8S1179	13	14	13	14	13	14	13	14
D3S1358	15	15	15	15	15	15	15	15
D6S1043	11	11	11	11	11	11	11	11
PENTAE	12	12	12	12	12	12	12	12
D19S433	15.2	15.2	15.2	15.2	15.2	15.2	15.2	15.2
PENTAD	8	13	8	13	8	13	8	13
